# Fixed Triple Therapy in Chronic Obstructive Pulmonary Disease and Survival. Living Better, Longer, or Both?

**DOI:** 10.1164/rccm.202003-0622ED

**Published:** 2020-06-15

**Authors:** Jørgen Vestbo

**Affiliations:** ^1^ Division of Infection, Immunity, and Respiratory Medicine The University of Manchester Manchester, United Kingdom and; ^2^ North West Lung Centre Manchester University NHS Foundation Trust Manchester, United Kingdom

Does regular pharmacological treatment including inhaled corticosteroids (ICS) reduce mortality in patients with chronic obstructive pulmonary disease (COPD)? Few questions have attracted more attention in COPD, and the number of commentaries, editorials, and reviews on this topic by far exceeds the limited number of original papers addressing the topic. However, here is one more.

In this issue of the *Journal*, Lipson and colleagues (pp. 1508–1516) report findings from a *post hoc* analysis of the IMPACT (Informing the Pathway of COPD Treatment) trial (1). The trial was enriched for exacerbators, and the primary outcome was the annual rate of on-treatment moderate and severe exacerbations comparing a fixed combination of fluticasone furoate (FF), umeclidinium (UMEC), and vilanterol (VI) with fixed combinations of UMEC/VI and FF/VI. Mortality was listed among “other efficacy outcomes” (2), and the article presents an expanded analysis of mortality compared with the one reported in their primary report (3). By obtaining information on vital status at end of the trial for an additional 574 patients, the authors had information on vital status for 10,313 of the 10,355 patients included in the trial. The authors compared mortality in patients randomized to FF/UMEC/VI with those randomized to either a fixed combination of FF/VI or a fixed combination of UMEC/VI. After 1 year, there were significantly fewer deaths in the triple-combination group (FF/UMEC/VI) than in the UMEC/VI group (absolute risk reduction [ARR], 0.83%; relative risk reduction [RRR], 28%; *P* = 0.042) but not when comparing the FF/VI group with the UMEC/VI group (ARR, 0.55%; RRR, 18%; *P* = 0.190). When analyses were restricted to on-treatment mortality, both these group analyses were statistically significant. The authors convincingly showed that missing outcome data on the 0.4% of the patient population were very unlikely to have had an impact on the reported estimates.

Only a few previous trials of an ICS-containing treatment have had mortality as a primary outcome. The TORCH (Toward a Revolution in COPD Health) study (4) found a 2.6% ARR and a 17.5% RRR in deaths when comparing the combination of salmeterol and fluticasone propionate with placebo. However, the *P* value was adjusted from 0.041 to 0.052 to take a late interim analysis into account, and the study has since been referred to as “negative.” The SUMMIT (Study to Understand Mortality and Morbidity) (5) compared a combination of vilanterol and fluticasone furoate with placebo in patients with moderate COPD at heightened cardiovascular risk and could not demonstrate an effect on overall mortality (ARR, 0.7%; RRR, 12%; *P* = 0.137). Observational studies had previously indicated a beneficial effect of ICS on mortality, but these are all open to criticism as they lack randomization (6). In neither TORCH nor SUMMIT did the long-acting β-agonist have any effect on mortality. In the UPLIFT (Understanding Potential Long-Term Impacts on Function with Tiotropium) trial, an effect on mortality, which was not the primary outcome, was likely present but depended on choice of analysis (7). No other single study of a fixed triple combination has analyzed mortality, but a recent pooled *post hoc* analysis indicated a favorable effect on mortality of the fixed combination of extrafine beclomethasone dipropionate, formoterol fumarate, and glycopyrronium bromide (8).

It is unlikely that there will be any more studies of inhaled medications with mortality as primary outcome. Even though treatment before entering a study is unlikely to affect the study findings (9), studies including patients who will have their existing treatment changed—and sometimes reduced—will always be open to criticism (10). Studies of symptomatic treatment-naive patients with COPD are not possible, and placebo-controlled trials are unethical. Withdrawal from longer-term studies is inevitable and will dilute study findings (11), and one could always question whether the findings from rigorous efficacy trials are transferable to usual clinical practice (12). Nonetheless, the IMPACT findings are probably the best we will have, and we cannot shy away from having an opinion on the effects seen, with the usual “more studies are needed.”

Now, should the discussion of these new IMPACT findings then focus on the small ARRs, the importance of the findings for the large patient group, the risk of pneumonia in the many versus the survival gain in the few, or the strengths and weaknesses of these findings? I would argue that this would be a waste of time. I would rather ask why we keep looking for reasons why a “proper” pharmacological treatment in COPD should *not* lead to a reduction in mortality in symptomatic COPD patients with a history of exacerbations. By “proper,” I mean a treatment that affects lung function, health status, and frequency of moderate and severe exacerbations—which we can achieve with long-acting bronchodilators in the majority of patients and with ICS in a proportion of these (13). To a COPD clinician it is clear that not all patients have marked benefits from the treatments, but to a vast number of patients, new long-acting inhaled drugs in simple combination inhalers have made a marked impact on their well-being and their ability to keep up with daily activities. Given what we know about risk factors for mortality in multimorbidity in general and COPD in particular, it really does not surprise me that these patients live longer. The challenge is now the same as for all other areas where individualized management is “the thing.”

How do we provide ICS-containing treatment to those who will benefit the most with the fewest side effects, in particular pneumonia? Blood eosinophils is undoubtedly a step in the right direction (13, 14), but better understanding and application of this and future biomarkers could help us better identify those with the biggest benefit. In the meantime, we can appreciate that for patients with COPD with frequent exacerbations we can already provide treatments that make them live both better and longer.

## Supplementary Material

Supplements

Author disclosures

## References

[1] Lipson DA Crim C Criner GJ Day NC Dransfield MT Halpin DMG *et al* IMPACT Investigators Reduction in all-cause mortality with fluticasone furoate/umeclidinium/vilanterol in patients with chronic obstructive pulmonary disease *Am J Respir Crit Care Med* 2020 201 1508 1516 32162970 10.1164/rccm.201911-2207OC PMC7301738

[2] Lipson DA Barnhart F Brealey N Brooks J Criner GJ Day NC *et al* Once-daily single-inhaler triple versus dual therapy in patients with COPD *N Engl J Med* 2018 378 1671 1680 29668352 10.1056/NEJMoa1713901

[3] Pascoe SJ Lipson DA Locantore N Barnacle H Brealey N Mohindra R *et al* A phase III randomised controlled trial of single-dose triple therapy in COPD: the IMPACT protocol *Eur Respir J* 2016 48 320 330 27418551 10.1183/13993003.02165-2015

[4] Calverley PM Anderson JA Celli B Ferguson GT Jenkins C Jones PW *et al* TORCH investigators Salmeterol and fluticasone propionate and survival in chronic obstructive pulmonary disease *N Engl J Med* 2007 356 775 789 17314337 10.1056/NEJMoa063070

[5] Vestbo J Anderson JA Brook RD Calverley PM Celli BR Crim C *et al* SUMMIT Investigators Fluticasone furoate and vilanterol and survival in chronic obstructive pulmonary disease with heightened cardiovascular risk (SUMMIT): a double-blind randomised controlled trial *Lancet* 2016 387 1817 1826 27203508 10.1016/S0140-6736(16)30069-1

[6] Collins R Bowman L Landray M Peto R The magic of randomization versus the myth of real-world evidence *N Engl J Med* 2020 382 674 678 32053307 10.1056/NEJMsb1901642

[7] Celli B Decramer M Kesten S Liu D Mehra S Tashkin DP UPLIFT Study Investigators. Mortality in the 4-year trial of Tiotropium (UPLIFT) in patients with chronic obstructive pulmonary disease *Am J Respir Crit Care Med* 2009 180 948 955 19729663 10.1164/rccm.200906-0876OC

[8] Vestbo J Fabbri L Papi A Petruzzelli S Scuri M Guasconi A *et al* Inhaled corticosteroid containing combinations and mortality in COPD *Eur Respir J* 2018 52 1801230 30209195 10.1183/13993003.01230-2018

[9] Vestbo J Dransfield M Anderson JA Brook RD Calverley PMA Celli BR *et al* Impact of pre-enrolment medication use on clinical outcomes in SUMMIT *ERJ Open Res* 2019 5 00203-2018 10.1183/23120541.00203-2018 PMC6387990 30815468

[10] Suissa S Ernst P Vandemheen KL Aaron SD Methodological issues in therapeutic trials of COPD *Eur Respir J* 2008 31 927 933 18216056 10.1183/09031936.00098307

[11] Vestbo J Anderson JA Calverley PM Celli B Ferguson GT Jenkins C *et al* Bias due to withdrawal in long-term randomised trials in COPD: evidence from the TORCH study *Clin Respir J* 2011 5 44 49 21159140 10.1111/j.1752-699X.2010.00198.x

[12] Woodcock A Boucot I Leather DA Crawford J Collier S Bakerly ND *et al* Effectiveness versus efficacy trials in COPD: how study design influences outcomes and applicability *Eur Respir J* 2018 51 1701531 29467200 10.1183/13993003.01531-2017

[13] Singh D Agusti A Anzueto A Barnes PJ Bourbeau J Celli BR *et al* Global strategy for the diagnosis, management, and prevention of chronic obstructive lung disease: the GOLD science committee report 2019 *Eur Respir J* 2019 53 1900164 30846476 10.1183/13993003.00164-2019

[14] Pascoe S Barnes N Brusselle G Compton C Criner GJ Dransfield MT *et al* Blood eosinophils and treatment response with triple and dual combination therapy in chronic obstructive pulmonary disease: analysis of the IMPACT trial *Lancet Respir Med* 2019 7 745 756 31281061 10.1016/S2213-2600(19)30190-0

