# Supplementary material for: Fixed Triple Therapy in Chronic Obstructive Pulmonary Disease and Survival. Living Better, Longer, or Both?
Source: Am J Respir Crit Care Med. 2020 Jun 15;201(12):1463–4. doi: 10.1164/rccm.202003-0622ED (PMC7301727; doi:10.1164/rccm.202003-0622ED)
Supplement: Supplements [file rccm.202003-0622ED.html]

Fixed Triple Therapy in Chronic Obstructive Pulmonary Disease and Survival. Living Better, Longer, or Both? | American Journal of Respiratory and Critical Care Medicine

- disclosures.pdf (146 KB)
